# Acute myocarditis after COVID-19 vaccination

**DOI:** 10.15537/smj.2022.43.11.20220292

**Published:** 2022-11

**Authors:** Sufana M. Saadi, Asseil A. Bossei, Loui K. Alsulimani

**Affiliations:** *From the Department of Emergency Medicine, King Abdulaziz University, Jeddah, Kingdom of Saudi Arabia.*

**Keywords:** myocarditis, pericarditis, COVID-19 vaccine, viral vaccines, coronavirus

## Abstract

Heart muscle inflammations were reported following SARS-CoV-2 messenger ribonucleic acid (RNA) vaccination by the Disease Control Centers in America, and cases of these inflammations reported as adverse effects of this COVID-19 vaccine application increased 1000 times since April 2021. A male individual, 18-year-old received vaccination with mRNA-1273 vaccine, and after a while attended the Emergency Department at King Abdulaziz University Hospital, Jeddah, Saudi Arabia. Upon presentation, the patient complained of a history of chest pain, and he had a high troponin level along with new-onset electrocardiogram changes. During his stay in hospital the patient’s blood circulation status remained stable, and no evidence of another infectious or immune cases was found. Although these vaccines are a must and very advantageous in fighting COVID-19 and their benefits are far beyond their risks, although it seems that there is a risk of myopericarditis cases. Under such conditions it is essential to rely on early diagnosis for control and deal with the possible cases of morbidity and mortality associated with these conditions.

Due to the appearance and spread of COVID-19 throughout the world a wide vaccination campaigns initiated worldwide. The first vaccine against SARS-CoV-2 was issued by the United State Food and Drug Administration on December 11, 2020.This vaccine works against infection and interaction with 2019 (COVID-19), that cause acute respiratory problems. The vaccines are manufactured by Pfizer, Moderna, and AstraZeneca, and each manufactured vaccine was recognized with special set of side effects. Also these vaccines had not been well tested, and for this reason emergency physicians were not able to take these side effects into consideration. Similar presentations can appear with infection and reaction of SARS-CoV-2, and both have possible complications.^1^ Pfizer-BioNTech was the first to distribute the vaccine BNT162b2 mRNA COVID-19 throughout the world. The vaccine has side effects including, headaches, fatigue, fever, muscle pain and other effects, such as ventricular dysrhythmia and hypertensive emergencies. The impact of immunization has not yet been proven by a sufficient number of articles. Notwithstanding the fact that case reports on the vaccines’ complications or side effects have been published, the vaccines are nevertheless provided, and vaccination is mandated in Saudi Arabia because it proved beneficial with minor risks.

All residents in Saudi Arabia are offered free health-care regarded as free vaccination against COVID-19. There are 494 hospitals and 22.5 beds per 10000 people, and 113000 physicians in 2019 in Saudi Arabia available under the use of Saudi health care center.^2,3^

The government formed a national committee under supervision of the Ministery of Health for facilitating effective response to COVID-19, across all sectors.^4^ This committee issues emergency measures to enhance this work. Within the health ministry the National Command and Control Center was activated to follow up the national, international, and regional updates and to obtain the appropriate actions.^5^ The committee has obtained early measures trying to stop and reduce spread of COVID-19, and these measures tackled the true and confirmed cases, calling back Saudi citizens from abroad, raising levels of procedures in Saudi ports, supply of the health sector with what it needs from materials and equipments, and evaluation of the risks of people groping.^5,6^ Since the early appearance and spread of COVID-19, Saudi Arabia issued warnings to its citizens not to travel to countries known to have high incidence of COVID-19. Also in March 15 and 21, 2020 and when COVID-19 reached high levels of spread the government banned all international and domestic flights.^7^

Cardiac complications are possible clinical scenarios. Previous articles noted that the high rate rise in mortality due to COVID-19 is associated with presence of high troponin levels.^8^ Myocarditis and pericarditis are the most serious complications. Myocarditis has been previously reported with other vaccinations. Heart muscle inflammation and myopericarditis may lead to death. The biological mechanism by which this occurs is still unknown. Both the European Medicines Agency and the Center for Disease Control and Prevention (CDC) are running research to study this connection using monitoring data. The first documented cases of myopericarditis after Pfizer vaccination originated at the end of April 2021.^9^ Since April 2021, there have been many reports reporting adverse cases of the vaccine system (VAERS) represented in pericarditis and myocarditis occurring after vaccination with mRNA COVID-19.^10^ In recently published articles, the diagnosis of myocarditis was carried out using noninvasive cardiac imaging and routine laboratory investigations, and more study is required to know the danger of myocarditis after vaccination.^10^ Therefore, patients who suffer chest pain or with a history of recent COVID-19 vaccination should be evaluated to be sure that they do not have cardiac involvement even if there is no active chest pain. It is recommended to continuously monitoring and reporting the vaccine adverse effects. Therefore, this case report describes and evaluates the complications that faced a young boy who received a COVID-19 vaccine and diagnosed with myocarditis/pericarditis.

## Case Report

We received in our Emergency Department (ED) at King Abdulaziz University Hospital, Jeddah, Saudi Arabia, a patient 18 years old on the 9^th^ of February 2022 with a history of chest pain. The patient has no previous heart problems.

### Clinical findings

He was complaining of a severe diffuse chest pain for 5 hours that prevented him from sleeping and there is change in breath and standing, without dyspnea or palpitation. He denied any recent viral illness. He never had history of any drug intake or previous surgeries. He felt these drawbacks only after receiving COVID-19 vaccination, and on the 5^th^ of February 2022, he received the 3^rd^ dose of mRNA-1273 vaccine. His first and second vaccination doses were Pfizer’s BioNtech COVID-19 mRNA-1273 vaccine. He denied experiencing similar presentation with the previous 2 doses. At presentation to the ED, the patient’s vital signs were stable, and he had no active chest pain; therefore, he was categorized as Canadian Triage and Acuity Scale priority 4 by our triaging system. His heart beat was normal without heart murmur or any additional heart sounds. No abnormalities were shown in the rest of the physical examination.

### Diagnostic testing

**Figure 1:**
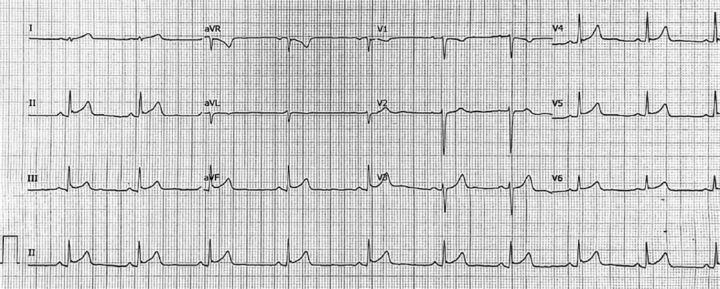
- Sinus rhythm electrocardiogram showed a PR depression in inferior threads and a persistent concave ST elevation without a reciprocal depression. aVR: augmented Vector Right, aVL: augmented Vector Left, aVF: augmented Vector Foot, V 1-6: precordial, or chest leads

**Figure 2:**
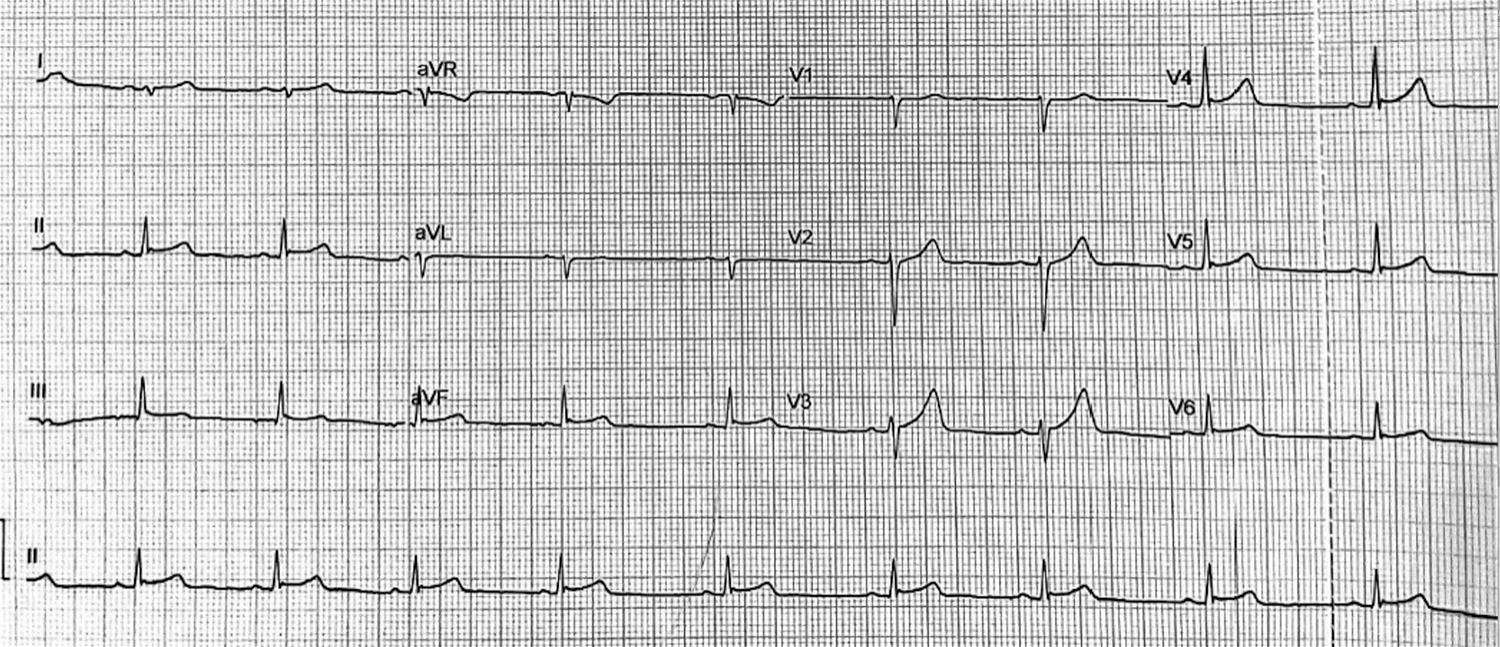
- Electrocardiogram on the 3^rd^ day of admission showing a normal paranasal sinuses rhythm with a normal ST segment in all threads with no atrioventricular conduction defects. aVR: augmented Vector Right, aVL: augmented Vector Left, aVF: augmented Vector Foot, V 1-6: precordial, or chest leads

Initial electrocardiogram (ECG) application demonstrated a normal paranasal sinuses rhythm with a PR depression and ST segment elevation in threads I, II, aVF, V5, and V6 with ST segment depression in V1 without reciprocal depression or atrioventricular conduction defects (Figure 1). Serial ECGs and troponin levels were ordered. The first troponin level was 16 µg/L (reference 0.02-0.04 µg/L), which started to increase gradually on the day of admission. The process of ECG was repeated on the 3^rd^ day of admission, and it resulted in normal paranasal sinuses rhythm and normal ST segment in all threads with no atrioventricular conduction defects (Figure 2). Myocarditis was established as the initial impression and diagnosis. Blood investigation results were as follows: white blood cells: 9.86 K/µL, hemoglobin: 15.7 g/dL, repeated troponin level: 18.29 µg/L, lactate dehydrogenase: 309 U/L, creatinine kinase (CK): 584 U/L, low-density lipoprotein: 1.69 mmol/L, CK-MB mass: 50.66 µg/L, and coagulation profile, within normal levels. Noninvasive cardiac imaging using transthoracic echocardiogram was carried out and showed normal left ventricle size and wall thickness with normal systolic and diastolic function. The left ventricular internal cavity size was normal. The pericardium had no evidence of pericardial effusion. The pleura had no evidence of pleural effusion. No significant mitral or aortic regurgitation was observed. Global left ventricular systolic function was normal, and visual estimation was 55-60% and chest X-ray was unremarkable (Figure 1).

### Therapeutic intervention

**Figure 3:**
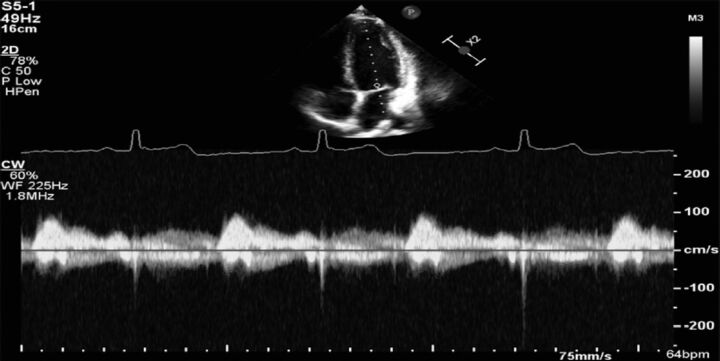
- Echocardiography on the first day of admission; left ventricle size and wall thickness.

Management was started with ibuprofen 600 mg orally, and the Cardiology Department was contacted for admission for further echocardiogram evaluation and investigations (Figure 3). The Cardiology Department admitted the patient as having myopericarditis with no hemodynamic or respiratory failure symptoms upon admission. Upon admission, the cardiology team started inpatient treatment with ibuprofen 400 mg 3 times a day for 4 days and initiated colchicine 500 mcg daily for 3 days.

### Follow-up and outcomes

**Figure 4:**
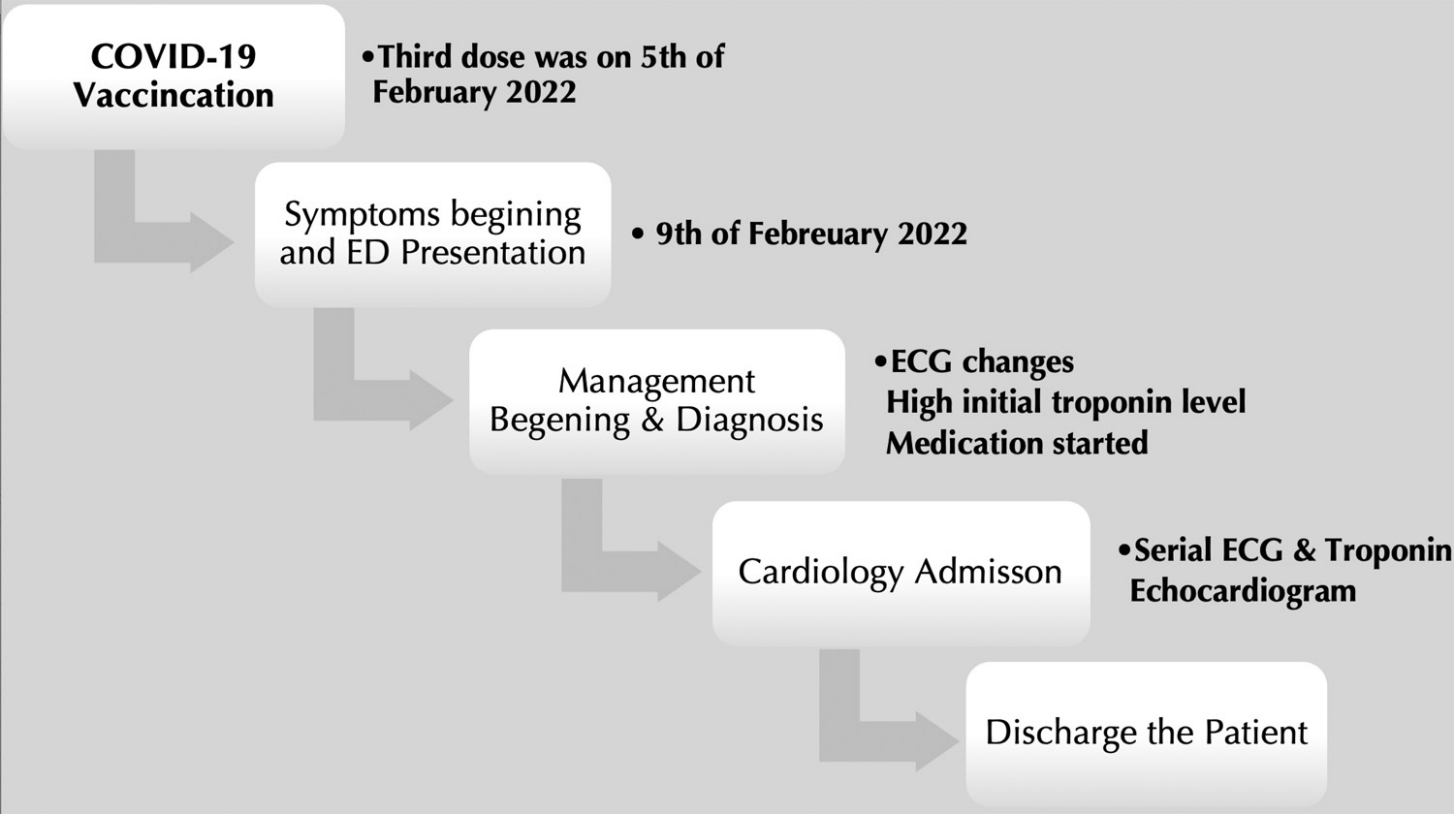
- Timeline of the patient’s stay. ED: Emergency Department, ECG: electrocardiogram

Throughout his stay in the hospital, the patient’s hemodynamic and respiratory statuses remained stable, without ventricular rhythm disorders, and without chest pain, and he recovered his complete health after one day of admission. Cardiac enzymes began to decrease after the initiation of symptom management. As a follow-up plan, the patient was discharged with an appointment with cardiology services and a cardiac magnetic resonance imaging (MRI) (Figure 4).

## Discussion

This case detected an unfortunate heart muscle inflammation post COVID-19 vaccination. Because mRNA technology is new to vaccine, its consequences cannot be predicted. Side effects of any vaccine, can not result from delayed local allergic reactions.^4^ Majority of patients (60%) who received the second dose of vaccine complained from a number of setbacks such as fever, headache, myalgia, and general malaise. Moderna reported only 1% of serious adverse cases in both the vaccinated and control groups. Adverse events considered serious if defined as death, a life-threatning, hospitalization, persistent or significant disruption of normal vital functions, or a congenital anomaly or birth defect. No thrombotic events were reported between the groups. After the first dose of the vaccine there were number of adverse effects particularly noticed in patients 49 years of age than >49 years, and these were commonly represented in site such as pain, fatigue, and headache. And in both age groups the injection site pain, fatigue, lethargy, headache, and tenderness were the most common adverse patient complain after the second vaccine dose. Individuals aged 49 years old were found to be more common with vaccination side effects for both doses.^5^

According to records in number of published articles throughout the world, the estimated incidence of diagnosed myocarditis appeared post COVID-19 vaccination treatment is 2.13 cases per 100,000 vaccinated individuals 42 days post the first dose of COVID-19 vaccine. The highest incidence was observed in males aged 16-29 years old. Based on the estimation of the CDC the incidence of heart muscle inflammation following a COVID-19 vaccination is 0.48 cases per 100,000, and 1.2 cases per 100,000 in vaccinated recipients between the ages of 18-29 years.^5^

Although the patient received Pfizer’s BioNtech mRNA-1273 vaccine in his first 2 doses, no similar appearance was documented at his third dose. Dionne at al11 analyzed a case series in the US involving 15 children under the age of 19 and followed them for adverse events, particularly myocarditis, after doses of Pfizer vaccine. Dionne at al11 found a likelihood that myopericarditis frequently occurs after the 2nd dose, while our patient’s condition occurred after receiving the Moderna vaccine and after the 3rd dose.^5^ The reason for this is unknown. Furthermore, Dionne at al11 mentioned that post-vaccination myopericarditis usually has a short-term course and the long-term course is not yet understood.5 Reviewing the literature, the incidence of myocarditis as a post-vaccination complication was higher in the Pfizer group (60%) than in the Moderna (33%) and Johnson & Johnson (7%) groups. However, Salah et al^7^ included adults in their study. Still, in 2021, Li et al^5^ carried out a study comparing Pfizer and Moderna vaccines and their association with myopericarditis. They found that the risk of developing myopericarditis was increased with the Pfizer vaccine.^5^

According to COVID-19 vaccination data of a Danish population, they discovered strong association between heart muscle inflammation and vaccination with mRNA-1273.^4^ A number of 48 and 21 individuals suffered myocarditis or myopericarditis within the first 28 days after vaccination with BNT162b2 and with mRNA-1273. Rate of myocarditis or myopericarditis increased significantly in those persons vaccinated with mRNA-1273 compared with unvaccinated individuals in follow-up.^4^ In our patient, we found that troponin levels were high, which increased the suspicion of myopericarditis 4 days after receiving the mRNA-01273 vaccine. In a population-based study carried out in Saudi Arabia, one in 2 participants was reported suffering at least one side effect within 14 days of the vaccination with BNT162b2 mRNA vaccine. The most common side effects were tenderness of injection-site, fatigue, and body ache with predominantly rapid onset, recovery, and rare risk for admission. In our region, we have very few published articles on the side effects of the BNT162b2 mRNA vaccine.^8^ The second vaccine dose witnessed more rate of side effects reaching up to 41% more than the first vaccine dose, irrespective of the interval between the doses. This result agrees with the report in the clinical trial which gave higher side effect rates (25-50% higher) and also agrees with a study carried out in the United Kingdom which gave 88% increase in side effects. The higher reactogenicity after the second dose of vaccination is associated with the first dose immunogenicity. Higher reactogenicity among COVID-19-recovering persons who received a SARS-CoV-2 vaccine was reported recently, probably because of elevated antibody titers, than among vaccine recipients who had never been naturally infected with SARS-CoV-2. A total of 7 participants complained of palpitations (6 had palpitations after the first dose, and one had palpitations after the second dose). Recently, acute pericarditis and myocarditis were reported a few hours after the second dose.^8^

In North America a number of 140 adults (<21 years old) were clinically suspected of incidents of myocarditis as a result of COVID-19 vaccination.^9^ Approximately 35% of the cases were confirmed by CDC as myocarditis after vaccination (49 confirmed, 91 probable) at 26 centers.^9^ Investigations revealed high troponin levels and cMRIs that met the Lake Louise criteria for myocarditis, and the rest were considered suspected cases. The clinical findings and symptoms were reached to, during the first week after vaccination and most patients have it after the second dose of vaccination. Most patients were male (90.6%) and White (66.2%). The complaint that most patients sufferred was chest pain (99.3%), and the patients were treated with nonsteroidal anti-inflammatory drugs.^9^ Another case summarized 7 healthy adolescent males suffering from chest pain within the first 4 days after the second COVID-19 vaccine dose, and they were all diagnosed with acute myocarditis or myopericarditis.^10^ The symptoms of 3 of these patients were treated by nonsteroidal anti-inflammatory drugs only, and 4 patients received corticosteroids and intravenous immunoglobulin.^10^ We conclude that the development of such a rare complication could be due to any of the mRNA vaccines.

In conclusion, individuals vaccinated with mRNA-1273 have been subjected to significantly increased rate of myopericarditis throughout the world, although the pathophysiology remains unclear. However, it is very important to vaccinate all people against COVID-19, mainly after it was confirmed that the benefits of vaccination outweigh its risks. Accordingly, the world is now engaged in carrying out large-scale vaccination campaigns against COVID-19, without considerations of possible risks such as heart muscle inflammation. Finally, it is essential to early recognize and manage the side effects of COVID-19 vaccination so that to decrease morbidity and mortality from such postvaccination complications.

## References

[1] Ministry of Health. The Kingdom of Saudi Arabia’s experience in health preparedness and response to COVID-19 pandemic. [Updated 2020; cited date 2020 Dec 6]. Available from: https://www.moh.gov.sa/en/Ministry/MediaCenter/Publications/Documents/COVID-19-NATIONAL.pdf, accessed 6 December 2020

[2] Ministry of Health. About Command And Control Center Riyadh: 2020. [Updated 2018 Jul 25; Cited 2020 Dec 25]. Available from: https://www.moh.gov.sa/en/CCC/about/Pages/default.aspx

[3] Peretto G , Sala S , Caforio ALP. Acute myocardial injury, MINOCA, or myocarditis? Improving characterization of coronavirus-associated myocardial involvement. Eur Heart J 2020; 41: 2124–2125. 32363406 10.1093/eurheartj/ehaa396 PMC7197551

[4] Schmitt P , Demoulin R , Poyet R , Capilla E , Rohel G , Pons F , et al. Acute myocarditis after COVID-19 vaccination: a case report. Rev Med Interne 2021; 42: 797–800. 34740463 10.1016/j.revmed.2021.10.003 PMC8523482

[5] Li M , Yuan J , Lv G , Brown J , Jiang X , Lu ZK. Myocarditis and pericarditis following COVID-19 vaccination: inequalities in age and vaccine types. J Pers Med 2021; 11: 1106. 34834458 10.3390/jpm11111106 PMC8624452

[6] Gargano JW , Wallace M , Hadler SC , Langley G , Su JR , Oster ME , et al. Use of mRNA COVID-19 vaccine after reports of myocarditis among vaccine recipients: update from the Advisory Committee on Immunization Practices - United States, June 2021. MMWR Morb Mortal Wkly Rep 2021; 70: 977–982. 34237049 10.15585/mmwr.mm7027e2 PMC8312754

[7] Salah HM , Mehta JL. COVID-19 vaccine and myocarditis. Am J Cardiol 2021; 157: 146–148. 34399967 10.1016/j.amjcard.2021.07.009 PMC8272967

[8] Menni C , May A , Polidori L , Louca P , Wolf J , Capdevila J , et al. COVID-19 vaccine waning and effectiveness and side-effects of boosters: a prospective community study from the ZOE COVID study. Lancet Infect Dis 2022; 22: 1002–1010. 35405090 10.1016/S1473-3099(22)00146-3 PMC8993156

[9] Larson KF , Ammirati E , Adler ED , Cooper LT Jr , Hong KN , Saponara G , et al. Myocarditis after BNT162b2 and mRNA-1273 vaccination. Circulation 2021; 144: 506–508. 34133884 10.1161/CIRCULATIONAHA.121.055913 PMC8340725

[10] Marshall M , Ferguson ID , Lewis P , Jaggi P , Gagliardo C , Collins JS , et al. Symptomatic acute myocarditis in 7 adolescents after Pfizer-BioNTech COVID-19 vaccination. Pediatrics 2021; 148: e2021052478. 34088762 10.1542/peds.2021-052478

[11] Dionne A , Sperotto F , Chamberlain S , Baker AL , Powell AJ , Prakash A , ET AL. Association of Myocarditis With BNT162b2 Messenger RNA COVID-19 Vaccine in a Case Series of Children. JAMA Cardiol 2021; 6: 1446–1450. 34374740 10.1001/jamacardio.2021.3471 PMC8356143

